# Volatile flavor characteristics of scallops (*Chlamys farreri*) with different drying methods were analyzed based on GC-IMS and GC-O-QTOF

**DOI:** 10.1016/j.fochx.2024.101960

**Published:** 2024-11-01

**Authors:** Pengfei Jiang, Xiaoqing Miao, Jing Li, Hang Qi, Shan Shang, Xiuping Dong

**Affiliations:** SKL of Marine Food Processing & Safety Control, National Engineering Research Center of Seafood, School of Food Science and Technology, Dalian Polytechnic University, Dalian 116034, China

**Keywords:** Scallop, Flavor, HS-GC-IMS, GC-O-QTOF, Drying

## Abstract

To investigate the effects of different drying methods on the flavor of scallops, headspace gas chromatography-ion mobility spectrometry (HS-GC-IMS) combined with gas chromatography-olfactometry-quadrupole time-of-flight (GC-O-QTOF) was used to analyze the flavor of scallops processed by five drying methods. A total of 62 volatile organic compounds (VOCs) were identified by GC-IMS. 27 characteristic aroma compounds were identified by GC-O-QTOF, and the highest content was trimethylamine. 17 aroma compounds with GC-O ≥ 6 were screened by the time-intensity method, and the maximum aroma intensity value of 2-acetyl-1-pyrroline was 9.9 aroma compounds with relative odor activity value (ROAV) ≥ 1 were identified. Using GC-O ≥ 6 and ROAV ≥1 as the criteria, 6 differential aroma compounds were further screened, and the results proved that naturally dried scallops had a heavier fishy odor. These findings will provide deeper insights into the processing of scallops.

## Highlights

• GC-IMS and GC-O-QTOF identified 62 and 27 volatile compounds, respectively.
• Naturally dried scallops have a strong fishy flavor.
• Screening of 17 aroma substances with GC-O ≥ 6 by time-intensity method.
• Screening of 6 differential aroma substances with GC-O ≥ 6 and ROAV ≥1.
• The maximum aroma intensity value of 2-Acetyl-1-pyrroline was 9.

## 1 Introduction

Scallop (*Chlamys farreri*) is a high-protein, low-fat shellfish, rich in a variety of nutrients and biologically active compounds with special health-care functions, such as taurine, choline, etc., with high food and medicinal value (Ding et al., 2022; Zhi et al., 2022). Studies have shown that regular consumption of scallops can promote growth and development and regulate blood sugar and blood pressure (Liu et al., 2022). However, the quality of scallops can deteriorate rapidly due to microbial, chemical and mechanical deterioration, and in order to effectively stop the growth of spoilage microorganisms and to reduce the rate of enzymatic reactions during storage, the moisture content should be controlled at a safe level (Shi et al., 2019). Drying is one of the main methods of intensive processing of scallops. The dried scallops are popular as a seafood ingredient in Asian cuisine because of their flavor and texture.

Natural drying, hot air drying, cold air drying, microwave drying, vacuum freeze drying, and other techniques have been used to dry scallop muscle (Xie et al., 2022； Yin et al., 2022). Natural drying (ND) has a long history, requires little need for expensive machinery or cutting-edge technology, and yields products with attractive color and excellent flavor. Natural drying does, however, come with significant drawbacks, including the inability to control the drying environment and the frequent ingestion of dust, mud, sand, and insects. The drying time is only practical in the autumn when the weather is dry with minimal rain and fewer insects like mosquitoes and flies. With the improvement of equipment, the hot air-drying technology came into being. Compared with natural drying, hot air drying (HAD) can shorten the drying time, so as to improve the drying efficiency (Cheng et al., 2021). However, hot air drying has some substantial disadvantages, including high energy consumption, and the potential for thermal damage if the temperature is not precisely controlled. Thermal damage can alter the texture, color, flavor, and nutritional value of products (Liu et al., 2023). In contrast, cold air drying (CAD) can avoid the problems arising from hot air drying and is widely used in the processing of aquatic products (Yu et al., 2021). Cold air drying positively impacts product quality and is effective in reducing heat-induced protein denaturation, lipid oxidation, color change and loss of flavor matter, but the drying process is relatively slow (Aykın-Dinçer & Erbaş, 2019; Zhu et al., 2023). Microwave drying (MWD) and vacuum freeze-drying (VFD) technologies are also frequently utilized in the processing of aquatic goods in addition to the above mentioned widely used techniques (Kipcak & Ismail, 2021). The process of microwave drying involves heating materials and preserving solid nutrition using electromagnetic radiation and may be the first technology with time- and energy-saving features that does not require direct contact between the heating medium and the product sample (Zaki et al., 2023). Vacuum freeze-drying refers to a method of drying by freezing the product under vacuum conditions and then sublimating to remove the water to achieve the purpose of drying, which is widely used in drying aquatic products, especially the preferred method for seafood rich in heat-sensitive compounds. Compared with other drying methods, vacuum freeze drying can well maintain the stability of nutrients (Guo et al., 2019).

As one of the essential indicators in measuring a product, the formation of flavor compounds was mainly induced by protein degradation, Maillard reaction, and lipid oxidation (Xuan et al., 2022). Headspace Gas Chromatography Ion Mobility Spectrometry (HS-GC-IMS) is a well-established technique for the determination of VOCs, which fully combines the high-resolution capability of GC technology with the high discrimination capability of IMS technology (Wang et al., 2020). HS-GC-IMS has been widely used for the detection and quantitative analysis of volatiles in food, food adulteration, and drug detection in recent years (Wu et al., 2024; Xiao et al., 2022). Zhu et al. (2022) characterized 30 VOCs in 5 different semi-dried *Takifugu obscurus* fillets by GC-IMS in order to investigate the effects of different drying techniques on the characteristic flavor of semi-dried *Takifugu obscurus* fillets. Therefore, to differentiate between the impact of various drying techniques on scallop flavor, HS-GC-IMS technology is employed. Gas chromatography-olfactometry (GC-O) is a method that combines gas chromatography detection methods with human sensory evaluation and is commonly used in the analysis and rating of flavor active ingredients. Currently, the GC-O technique is commonly used for the determination of characteristic flavors in meat, aquatic products, flavored items, and alcohol.

This study focused on how different drying methods affect the flavor of scallops. GC-IMS technique was used to construct fingerprints of VOCs in scallops and analyze the differences in volatile constituents in scallops treated with different drying methods, which were combined with GC-O-QTOF to identify the key flavor presenting compounds in scallops. The study's findings can serve as a guide for the development of novel techniques for drying scallops and have a reference value for identifying the characteristic flavor compounds in scallops.

## 2 Materials and methods

### 2.1 Samples and chemicals

Each autumn is the harvesting season for ctenophore scallops from Dalian, China, when the scallops are at their fattest and best enjoyed. Fresh ctenophore scallops (diameter 6.31 ± 0.40 cm; weight 29.35 ± 3.66 g) were caught on 20 October 2022 in the Bohai Sea. After transportation to the shore, the shells were opened by skilled workers using special tools to remove the skirt, gills, gonads, and visceral masses, and then the scallop adductor muscles were taken for subsequent experiments. The removed scallop adductor muscles were placed in sealed plastic bags in an insulated box with ice and transported to the laboratory within a span of 60 min. The samples were cooked for 8 min in 3 *V*/V salted water at the ratio of 1:2 (based on weight), then drained and let cool to room temperature.

The drained samples were divided into 5 groups: natural drying (ND), hot air drying (HAD), vacuum freeze drying (VFD), microwave drying (MWD), and cold air drying (CAD). Five groups of scallops were dried to control the final moisture content to 19 % - 20 %. Natural drying was laying the samples evenly placed on a layer of gauze, and covered with another layer of gauze to prevent insect infestation and dust contamination. Subsequently, they were dried at natural conditions for 96 h. Hot air drying and cold air drying involved placing the samples in a drying oven set at 40 °C and 20 °C, respectively, for 48 h and 96 h of drying time. Vacuum freeze drying involved drying a sample for 96 h using a vacuum freeze dryer (LG1.5, Xinyang, China) with a cold trap temperature of −40 °C, a vacuum of 10 Pa, and a heating temperature of 20 °C. Microwave drying was the use of a microwave oven to control the moisture content of the sample to 19 % - 20 %.

n-ketones (C4–C9) were purchased from Sinopharm Chemical Reagent Co., Ltd., Shanghai, China and n-alkanes (C7-C40, ≥ 97 %) were purchased from Anpu Experimental Technology Co., Ltd., Shanghai, China.

### 2.2 HS-GC-IMS analysis

VOCs in scallops were analyzed using HS-GC-IMS (FlavourSpec ®, Dortmund, Germany), with a slight modification of the method described by Xie et al. (2023). The scallop adductor muscles were crushed and accurately weighed 1 g into a 20 mL headspace bottle, added with 3 mL water, incubated at 60 °C and 500 rpm for 5 min, then inject 500 μL into the injector at 85 °C. High-purity nitrogen was used to drive the samples into a chromatographic column (MXT-5, 15 m × 0.53 mm × 1.0 μm, Restek Corporation, USA) which was kept at 60 °C. The 99.99 % nitrogen gas was considered as a transmission medium following the programmed speed by 2 mL/min for 2 min, 10 mL/min for 8 min, 100 mL/min for 10 min, and 150 mL/min for 10 min. The mixture gas was ionized in the IMS ionization cell. Retention indices (RI) for individual volatile chemicals were calculated using n-ketones C4–C9 as reference standards. The RI and drift time (DT) of VOCs were compared to those of standard chemicals using collations from the instrumental database (FlavorSpec®, Germany) and RI. The height or peak area was defined via the signal intensity.

### 2.3 Solid phase microextraction of aroma compounds (SPME)

The five kinds of dried scallop samples were taken separately and fully ground with a hybrid grinder (Retsch MM 400) under the grinding condition of 30 Hz for 1.5 min and 200 mg of powder was weighed accurately and put into 15 mL sample bottles for reserve. The SPME Arrow (DVB/CWR/PDMS 1.1 mm × 120 μm, Palsystem, Switzerland) underwent a 30-min aging process at 250 °C. Following this, the samples were incubated at 60 °C for 20 min, after which the extraction head was introduced into the sample vials for the extraction process. The extraction was carried out at 60 °C for a duration of 40 min. Subsequently, the sample was desorbed at 250 °C for 3 min and finally aged for an additional 15 min at the same temperature.

### 2.4 GC-QTOF analysis

VOCs were analyzed and identified by GC-QTOF (7890B-7200, Agilent, American). Gas chromatography column HP-5MS UI (30 m × 0.25 mm × 0.25 μm), non-split injection, carrier gas He, column flow rate 1.5 mL/min, warming program: 40 °C start (2 min), 2.5 °C/min up to 105 °C, 30 °C/min up to 230 °C (3 min). EI (Electron impact) source was with an ionization energy of 70 eV, an ion source temperature of 230 °C, a quadrupole temperature of 150 °C, and a mass scan range of 50 to 600 *m*/*z*.

### 2.5 GC-O analysis

Chromatographic conditions and heating procedures were consistent with the GC–MS conditions in Section 2.4. The effluent entered the mass spectrometer and the sniffing device (ODP4, Gerstel, Germany) at a 1:1 split ratio. The olfactory experiments were carried out by three professional sensory evaluators (one man and two women) in a tasting room with 25 ± 2 °C temperature, 50 % – 60 % relative humidity, and wind-free fresh air. The experiment employed the time-intensity method, with odor intensity assessed on a 4-point scale ranging from 0 (undetectable) to 4 (strong). Aromatically active compounds in five scallop species were evaluated sequentially, the characteristics of the odors smelled and their intensity were described and recorded, and the final value of the olfactory aroma intensity of each substance was used to describe the contribution of the substance to the aroma.

Note: Dalian Polytechnic University did not require ethical issues in performing this experiment, and all participants participated voluntarily and signed a right to information.

### 2.6 Qualitative analysis

VOCs in 15 scallop samples (3 × 5 groups) were qualitatively analyzed by a combination of mass spectrometry (MS), retention index (RI), and olfaction. Additionally, specific essential aroma compounds were characterized by comparing them against standards. MS results were analyzed using the Agilent MassHunter Unknowns software (Agilent Technologies Inc., U.S.A.) and NIST 17 mass spectrum database (National Institute of Standards and Technology, U.S.A.), and those with a match of ≥70 were selected as the identified compounds; retention index (RI): the series alkanes from C7 to C30 were used as a reference, and the actual retention indices were calculated for each compound based on the peak times of the targets and the peak times of the series of alkanes. The actual retention indices of each compound were calculated and analyzed in comparison with the theoretical retention indices reported in the literature (Zhao et al., 2022).

### 2.7 Statistical analysis

At least three independent replications of each experiment were performed. Using the SPSS Statistics 20.0 program (IBM, Armonk, NY, USA), a one-way analysis of variance (ANOVA) was used to examine the data. To compare the data for significance (*P* < 0.05), the Duncan's test was used. The data was displayed as mean ± SD. Histograms were generated using GraphPad Prism, while principal compound analysis (PCA) was conducted using MetaboAnalyst, and heatmaps were constructed using ChiPlot.

## 3 Results and discussion

### 3.1 HS-GC-IMS analysis of scallops treated by different drying methods

#### 3.1.1 HS-GC-IMS topographic plots analysis of scallops treated by different drying methods

**Fig. 1:**
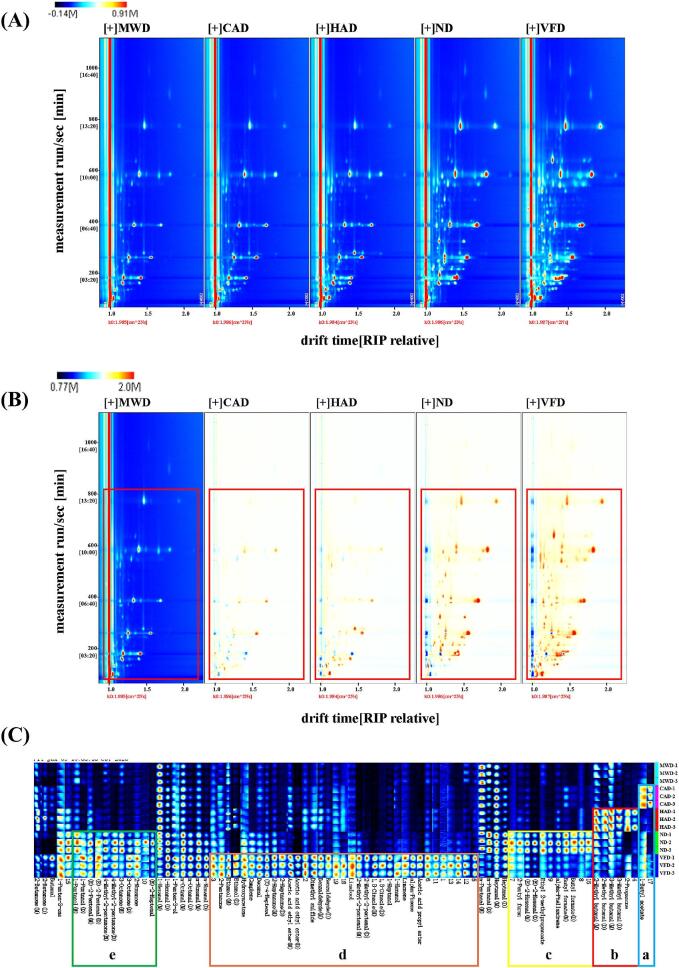
2D-topographic plots (A: vertical view; B: difference view), Fingerprints (C) of VOCs in scallops with different drying methods.

VOCs in scallops treated by different drying methods were analyzed by HS-GC-IMS, and two-dimensional spectra of VOCs in scallops were obtained, as shown in Fig. 1A. In the spectrogram, red vertical lines denote RIP peaks (normalized reactive ion peaks), with each point representing a volatile compound. Colors indicate the concentration of the substance; red signifies higher concentration, while white denotes lower concentration. The concentration of the volatile compound increases with darker color. The spectra showed that the retention times of most VOCs ranged from 100 to 800 s, and the drift times were 1.0–2.0 ms, indicating that there were more active VOCs in the region and that most of VOCs in the five scallops were similar. In order to compare the differences in a concise manner, a difference comparison model was used, with MWD as the reference and the other four scallops as the deducted reference spectra; the background was white after deduction. Red indicates higher volatile flavor substance content compared to the reference, while blue indicates lower content., and the results were shown in Fig. 1B. In comparison to MWD, HAD and CAD exhibited fewer red and blue signals, while ND and VFD showed a higher number of red signals, suggesting minor differences between microwave-dried, hot-air-dried, and cold-air-dried scallops, but significant disparities with naturally-dried and vacuum-freeze-dried scallops. Additionally, the content of VOCs indicated by red spots was higher in ND and VFD. Vacuum freeze-dried treated scallops could maximize the retention of active ingredients in scallops, maintain flavor and reduce aroma loss due to shorter exposure to air. In contrast, naturally dried scallops exhibited greater variation in volatile compound content due to the complexities of the natural environment.

#### 3.1.2 *Construction of fingerprints of scallop*s *prepared by different drying methods*

Fingerprint spectra of VOCs in scallops processed by different drying methods were generated using the Gallery Plot plug-in configured for GC-IMS, as shown in Fig. 1C. In the figure, each row represents all the signal peaks of volatiles obtained from a single scallop sample, and each column shows the signal peaks of the same volatile compound contained in different samples, with a darker color of red signals a mass concentration of the substance (Yao et al., 2022). A visual comparison of the differences in VOCs in each sample can be seen in Fig. 1C, where all four scallop samples had relatively independent characteristic flavor regions, except for MWD, which did not have its characteristic flavor region. The complexity of the volatile flavor compounds in the VF suggested that vacuum freeze-drying can be a good way of retaining the flavors of the Scallops and generating new flavor compounds. Butyl acetate may be an important indicator of the flavor of aquatic products with pleasant fruity odors (Takagi et al., 2020) and it was the most abundant substance in CAD (Fig. 1C-a), presuming that cold-air drying could have imparted a stronger fruity aroma to Scallops. The most abundant volatile constituents of HAD include primarily 2-methylbutanal, 3-methylbutanal, and 2-pentanone (Fig. 1C-b). 2-methylbutanal and 3-methylbutanal have a nutty aroma and are the products of a Maillard reaction. Kitajima et al. (2023) showed that 3-methylbutyraldehyde was a good freshener. 2-pentanone provided some fruity notes to the scallops, but due to its high threshold (300 μg/kg), it contributed less to the overall flavor of the scallops. Butyl formate (sweet, fruity), 2-Pentyl furan (greasy, fruity) and Ethyl 2-Methylpropanoate (sweet, fruity) are volatile constituents (Fig. 1C-c) with high content in ND, suggesting that natural drying may provide some fruity and greasy aroma to scallops. The most abundant compounds in VFD are mainly propyl acetate, alpha-Pinene, hydroxyacetone, etc. (Fig. 1C-d). Wang et al. (2023). also confirmed that propyl acetate and alpha-Pinene are the main odorants in vacuum freeze-dried samples.

#### 3.1.3 Qualitative analysis of scallops treated by different drying methods

The results of the qualitative analysis of volatile flavor compounds by comparing RI, RT and DT from NIST 2014 and GC-IMS libraries were shown in Table 1. A total of 81 volatile flavor compounds were detected by GC-IMS, of which a total of 62 VOCs could be explicitly characterized (monomers and dimers of some compounds), mainly including 26 aldehydes, 10 alcohols, 13 ketones, 7 esters, 4 terpenes, 1 sulfide, and 1 furan.

**Table 1:** Qualitative analysis of VOCs in scallops treated by different drying methods.

Count	Compound	CAS#	Formula	RI	Rt [*sec*]	Dt [a.u.]	Comment
1	Decanal	112–31-2	C_10_H_20_O	1187.2	989.453	1.54089	
2	n-Nonanal	124–19-6	C_9_H_18_O	1103.8	772.529	1.46513	Monomer
3	n-Nonanal	124–19-6	C_9_H_18_O	1103.4	771.637	1.94534	Dimer
4	2-Nonanone	821–55-6	C_9_H_18_O	1095.9	754.677	1.4057	
5	1,8 Cineole	470–82-6	C_10_H_18_O	1041.8	642.642	1.29172	Monomer
6	1,8 Cineole	470–82-6	C_10_H_18_O	1040.1	639.488	1.73043	Dimer
7	n-Octanal	124–13-0	C_8_H_16_O	1010.2	585.246	1.39737	Monomer
8	n-Octanal	124–13-0	C_8_H_16_O	1009.9	584.615	1.82712	Dimer
9	3-Octanone	106–68-3	C_8_H_16_O	989.3	546.772	1.30604	Monomer
10	3-Octanone	106–68-3	C_8_H_16_O	989.6	547.403	1.71252	Dimer
11	1-Heptanol	111–70-6	C_7_H_16_O	987.5	542.988	1.39379	
12	2-Pentyl furan	3777-69-3	C_9_H_14_O	992.9	554.341	1.2577	
13	Benzaldehyde	100–52-7	C_7_H_6_O	975.7	519.216	1.15327	Monomer
14	Benzaldehyde	100–52-7	C_7_H_6_O	975.7	519.216	1.475	Dimer
15	(*E*)-2-Heptenal	18,829–55-5	C_7_H_12_O	959.5	488.432	1.25796	
16	(Z)-4-heptenal	6728-31-0	C_7_H_12_O	896.7	384.997	1.15425	
17	Heptanal	111–71-7	C_7_H_14_O	898.0	386.976	1.33541	Monomer
18	Heptanal	111–71-7	C_7_H_14_O	897.0	385.393	1.70529	Dimer
19	2-Heptanone	110–43-0	C_7_H_14_O	887.2	371.939	1.25993	Monomer
20	2-Heptanone	110–43-0	C_7_H_14_O	885.9	370.356	1.63484	Dimer
21	1-Hexanol	111–27-3	C_6_H_14_O	875.3	356.901	1.3178	
22	(E)-2-Hexenal	6728-26-3	C_6_H_10_O	848.7	325.449	1.18555	Monomer
23	(E)-2-Hexenal	6728-26-3	C_6_H_10_O	847.1	323.723	1.52026	Dimer
24	1-hexanal	66–25-1	C_6_H_12_O	785.2	261.107	1.26453	Monomer
25	1-hexanal	66–25-1	C_6_H_12_O	784.6	260.587	1.56321	Dimer
26	4-Methyl-2-pentanone	108–10-1	C_6_H_12_O	772.5	248.372	1.17228	Monomer
27	4-Methyl-2-pentanone	108–10-1	C_6_H_12_O	771.2	247.073	1.481	Dimer
28	1-Pentanol	71–41-0	C_5_H_12_O	769.3	245.254	1.25266	Monomer
29	1-Pentanol	71–41-0	C_5_H_12_O	769.0	244.994	1.5148	Dimer
30	(E)-2-Pentenal	1576-87-0	C_5_H_8_O	747.2	224.562	1.11316	Monomer
31	(E)-2-Pentenal	1576-87-0	C_5_H_8_O	748.0	225.265	1.36132	Dimer
32	n-pentanal	110–62-3	C_5_H_10_O	695.9	183.065	1.19139	Monomer
33	n-pentanal	110–62-3	C_5_H_10_O	692.0	180.251	1.42189	Dimer
34	acetic acid propyl ester	109–60-4	C_5_H_10_O_2_	705.4	190.098	1.47994	
35	2-Pentanone	107–87-9	C_5_H_10_O	676.1	171.987	1.12241	
36	2-methyl Butanal	96–17-3	C_5_H_10_O	662.0	165.657	1.1712	Monomer
37	2-methyl Butanal	96–17-3	C_5_H_10_O	661.2	165.305	1.40002	Dimer
38	3-Methyl butanal	590–86-3	C_5_H_10_O	641.1	156.689	1.17709	Monomer
39	3-Methyl butanal	590–86-3	C_5_H_10_O	649.0	160.03	1.4118	Dimer
40	acetic acid ethyl ester	141–78-6	C_4_H_8_O_2_	598.3	139.823	1.11449	Monomer
41	acetic acid ethyl ester	141–78-6	C_4_H_8_O_2_	601.9	141.189	1.34048	Dimer
42	2-Butanone	78–93-3	C_4_H_8_O	580.7	133.45	1.07096	Monomer
43	2-Butanone	78–93-3	C_4_H_8_O	586.5	135.498	1.24972	Dimer
44	Butanal	123–72-8	C_4_H_8_O	594.0	138.23	1.29881	
45	Dimethyl sulfide	75–18-3	C_2_H_6_S	524.8	115.013	0.97371	
46	2-Propanone	67–64-1	C_3_H_6_O	497.8	107.047	1.12561	
47	Ethanol	64–17-5	C_2_H_6_O	469.6	99.308	1.05244	Monomer
48	Ethanol	64–17-5	C_2_H_6_O	461.8	97.26	1.13579	Dimer
49	1-Penten-3-ol	616–25-1	C_5_H_10_O	678.0	172.859	0.95032	
50	Hydroxy acetone	116–09-6	C_3_H_6_O_2_	636.6	154.829	1.0398	
51	1-Penten-3-one	1629-58-9	C_5_H_8_O	678.8	173.243	1.07979	
52	1-butyl acetate	123–86-4	C_6_H_12_O_2_	801.4	276.258	1.23923	
53	Limonene	138–86-3	C_10_H_16_	1036.4	632.38	1.21633	
54	alpha-Phellandrene	99–83-2	C_10_H_16_	1004.8	575.917	1.22381	
55	2-Methyl-2-pentenal	623–36-9	C_6_H_10_O	822.6	297.371	1.16646	Monomer
56	2-Methyl-2-pentenal	623–36-9	C_6_H_10_O	823.3	298.031	1.49945	Dimer
57	Ethyl 2-methylpropanoate	97–62-1	C_6_H_12_O_2_	752.1	229.027	1.19274	
58	Butyl formate	592–84-7	C_5_H_10_O_2_	739.0	217.343	1.21571	Monomer
59	Butyl formate	592–84-7	C_5_H_10_O_2_	737.6	216.131	1.50946	Dimer
60	Linalool	78–70-6	C_10_H_18_O	1103.6	771.942	1.22286	
61	alpha-Pinene	80–56-8	C_10_H_16_	929.8	436.395	1.23051	
62	Camphene	79–92-5	C_10_H_16_	945.0	462.268	1.21478	

**Fig. 2:**
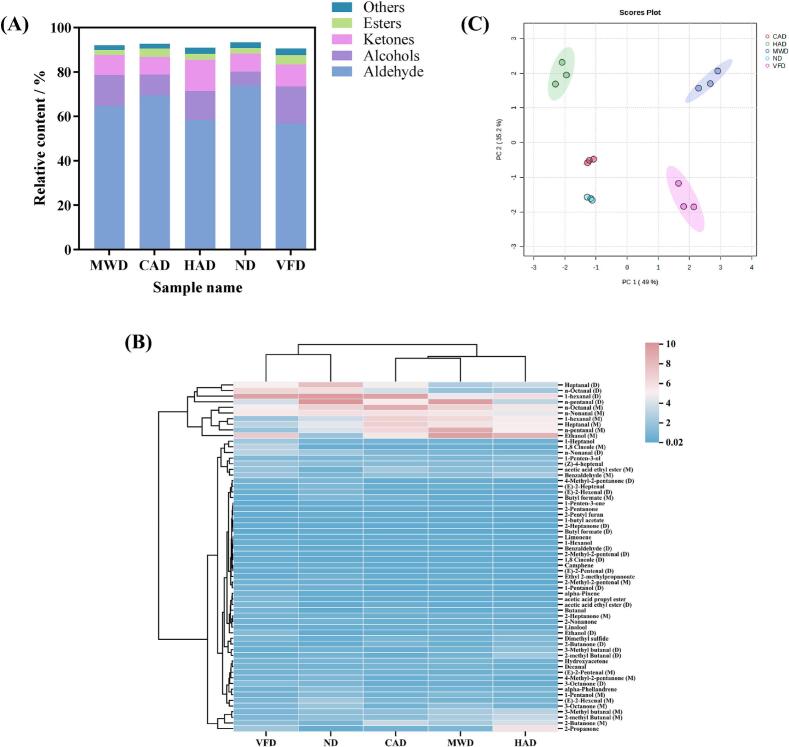
Stacking diagram of the percentage of each compound (A), Heatmap of VOCs in scallops with different drying methods (B), PCA analysis of VOCs in scallops with different drying methods (C).

To more clearly illustrate how each volatile ingredient contributes to the sample's overall flavor, the percentage stacking of VOCs was plotted as shown in Fig. 2A. Aldehydes dominated the volatile flavor compounds in the five scallop species, comprising 50 %–70 % of the total content. Aldehydes mainly originate from lipid oxidation and degradation and were important flavor compounds in scallops due to their low threshold and high content (Zeng et al., 2020). VFD had the lowest aldehyde concentration whereas ND had the highest, which showed that natural drying can retain aldehydes well. The next higher contents were alcohols and ketones, which were about 10 %. Alcohols can be formed by enzymatic oxidative decomposition of unsaturated fatty acids and reduction of carbonylamines (Guo et al., 2018). There were significant differences in the content of alcohols in scallops treated by different drying methods, especially in ND where the content of alcohols was the lowest, mainly due to the fact that the content of ethanol in natural drying was significantly lower than that of ethanol in other drying methods. Ketones are generally produced through amino acid catabolism and thermo-oxidative degradation of polyunsaturated fatty acids (Xu et al., 2023). Ketones were higher in HAD-treated scallops, which was mainly due to the higher content of acetone. The higher content of acetone may be due to the fact that some organic acids are easily decarboxylated into ketones when subjected to uneven heat (Sun et al., 2023).

VOCs in scallops treated by different drying methods were further analyzed based on the results of visual hierarchical clustering of heat maps, see Fig. 2B. 5 scallop samples were classified into two main groups according to the vertical pattern, one for VFD and ND, and the other for CAD, MWD, and HAD. All VOCs in the 5 scallop samples were broadly classified into 3 regions. 1-hexanal (D) was higher in VFD to enhance the fruity aroma. 1-hexanal (D) and n-pentanal (D) were higher in ND to enhance the fruity aroma. Hexanal (D) was higher in VFD to enhance the fruity aroma. 1-hexanal (D) and n-pentanal (D) were higher in ND. 1-hexanal (D) and n-Octanal (M) were higher in CAD. n-pentanal and Ethanol (M) were higher in MWD. n-Pentanal and Ethanol (M) were higher in HAD. The content of Ethanol (M) was higher in HAD.

#### 3.1.4 PCA analysis

In order to emphasize the differences in aroma characteristics between scallops treated by different drying methods, principal compound analysis (PCA) was carried out on scallops treated by different drying methods, and the results were shown in Fig. 2C, with a contribution of 49 % for PC1 and 35.2 % for PC2, and a cumulative contribution of 84.2 % for the two. MWD, CAD and HAD occupy one end of the PCA, while ND and VFD occupy the other, indicating that the VOCs in MWD, CAD and HAD were relatively similar, while those in ND and VFD were more varied. The largest difference between MWD and VFD among the five scallop samples was mainly due to the fact that microwave drying promotes the Maillard reaction, whereas vacuum freeze-drying inhibits the Maillard reaction, which led to a large flavor difference between them. The results of the PCA analyses visualized the differences in flavor between scallops treated with different drying methods.

### 3.2 HS-SPME-GC-O-QTOF analysis of scallops treated by different drying methods

#### 3.2.1 Statistical classification analysis of VOCs

**Fig. 3:**
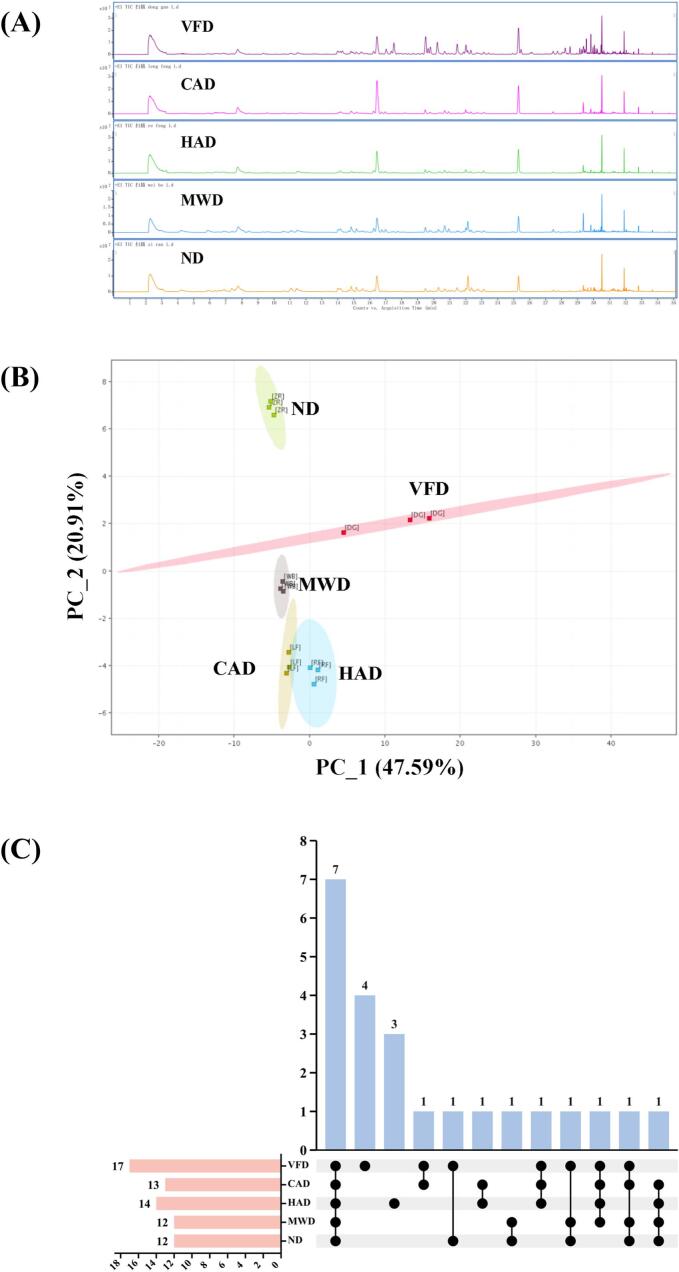
Total ion chromatograms of scallops with different drying methods (A), PCA scores of VOCs in scallops with different drying methods (B), Upset (C).

HS-SPME/GC-TOFMS was used to obtain the total ion flow diagrams of the five scallops treated by different drying methods, as shown in Fig. 3A. There were obvious differences in the peak intensities and the number of peaks in the color chromatograms of the five scallops, and the main peaks of the five samples were concentrated in 14–32 min. As shown in Fig. 3B, the volatile aroma compounds in scallops were analyzed by principal compounds. ND and VFD were at the top of the PCA coordinate axes, while MWD, HAD and CAD were at the bottom of the coordinate axes, and the three of them were close to each other and far away from ND and VFD. It was found that the volatile aroma compounds in ND and VFD differed significantly from those in MWD, HAD and CAD, which was in agreement with the results of GC-IMS.

#### 3.2.2 Characteristic aroma compound analysis

The olfactory analysis of scallops treated with different drying methods using a combination of GC-O and GC-QTOF resulted in 27 aroma compounds that could be clearly characterized, as shown in Table 2. As shown in Fig. 3C, 17, 13, 14, 12 and 12 aroma compounds were found in VFD, CAD, HAD, MWD and ND, respectively. Seven aroma compounds were found in five scallop species, namely methylamine, N, N dimethyl-, Acetic acid, heptanal, pyrazine, 2,5-dimethyl-, 1-octen-3-ol, 5-hepten-2-one, 6-methyl- and pyrazine, 2,6-diethyl-. Combined with Table 2, it could be seen that trimethylamine was highest in five scallop species, especially in MWD and ND, which were much higher than the trimethylamine content in the other three scallops. Trimethylamine, the main amine compound in aquatic products, is produced by the reduction of trimethylamine oxides by parthenogenetic anaerobic bacteria, which has a very strong ichthyophagous odor with a low sensory threshold (Yang et al., 2024), and usually affects the overall ichthyophagous odor of scallops, so that the fishy odors were greater in MWD and ND. There were four unique characteristic aroma compounds in VFD, which were Benzaldehyde, 2, 4-heptadienal, (E, E)-, 2-nonanone, and 3-cyclohexene-1-methanol. Three unique characteristic aroma compounds in HAD, which were o-xylene, ethanone, 1-(1H-pyrrol-2-yl)-, and pyrazine, 3, 5-diethyl-2-methyl-. 1-(1H-pyrrol-2-yl)- and pyrazine, 3, 5-diethyl-2-methyl-. In contrast, CAD, MWD and ND did not have their unique characteristic aroma compounds.

**Table 2:** Characteristic flavor compounds in scallop samples of different drying methods.

Compound	CAS	Taste Description	RI	RI	VFD		HAD		CAD		MWD		ND	
					Strength	Relative content (%)	Strength	Relative content (%)	Strength	Relative content (%)	Strength	Relative content (%)	Strength	Relative content (%)
Methylamine, N, N- dimethyl-	75–50-3	Shredded squid	/	502	8	9.24	8	9.96	7	10.96	8	22.45	7	25.14
Dimethyl sulfide	75–18-3	Pale, Salty, Pyridine	/	520	3	0.65	0	/	2	1.37	0	/	0	/
Acetic acid	64–19-7	sour	/	610	4	0.87	6	2.62	5	2.19	1	0.53	4	1.03
2,3-Butanediol	513–85-9	unpleasant smell, paint, garbage	767	788	6	0.25	4	1.05	3	0.54	0	/	0	/
Hexanal	66–25-1	grass	793	800	1	0.46	0	/	0	/	0	/	2	0.87
Oxazole, trimethyl-	20,662–84-4	perfume	851	852	0	/	1	0.00	0	/	0	/	0	/
Benzene, 1,3-dimethyl-	108–38-3	Tide, pills, herbal, seasonings	863	866	0	/	0	/	0	/	6	0.52	5	0.28
o-Xylene	95–47-6	fragrant	889	887	0	/	2	0.03	0	/	0	/	0	/
Heptanal	111–71-7	Shredded Squid, Fishy, rubber, Plastic	900	901	5	0.28	5	0.11	7	0.22	7	0.99	6	1.53
Pyrazine, 2,5-dimethyl-	123–32-0	Potatoes, savory aroma, salty aroma, wet flour, sweet potatoes	909	917	6	0.14	5	0.45	7	0.08	7	0.14	8	0.18
2-Acetyl-1-pyrroline	85,213–22-5	Cooked rice	922	922	9	/	8	/	9	/	5	/	6	/
Benzaldehyde	100–52-7	sweet and bitter	957	962	2	1.70	0	/	0	/	0	/	0	/
Dimethyl trisulfide	3658-80-8	Fishy, pungent, air-dried small crabs	965	970	7	0.15	5	0.16	2	0.01	3	0.06	0	/
1-Octen-3-ol	3391-86-4	Rust, paint	979	980	8	0.90	3	0.51	5	0.95	7	1.79	5	2.30
5-Hepten-2-one, 6-methyl-	110–93-0	Hawthorn Candy, Hawthorn, Fragrance	986	986	8	0.17	6	0.03	7	0.04	5	0.03	4	0.03
Pyrazine, 2-ethyl-6-methyl-	13,925–03-6	light Cooked rice	1001	1003	0	/	4	0.27	2	0.01	0	0.00	0	/
Octanal	124–13-0	Fresh, fragrant	1004	1003	5	0.64	0	/	0	/	8	2.22	5	3.39
2,4-Heptadienal, (E, E)-	4313–03-5	Fresh, slightly sweet and smelly	1011	1012	5	0.04	0	/	0	/	0	/	0	/
Ethanone, 1-(1H-pyrrol-2-yl)-	1072–83-9	snacks、plum、sweet	1062	1064	0	/	8	1.02	0	/	0	/	0	/
Pyrazine, 2,6-diethyl-	13,067–27-1	minty、almond	1080	1084	6	0.02	5	0.14	4	0.11	7	0.07	5	0.07
Pyrazine, tetramethyl-	1124-11-4	Coffee bean、chocolate biscuit、walnut、	1088	1089	4	0.00	8	0.10	2	0.10	6	0.08	6	0.02
2-Nonanone	821–55-6	fragrant、neutral	1094	1092	5	0.55	0	/	0	/	0	/	0	/
Ethanone, 1-(4,5-dihydro-2-thiazolyl)-	29,926–41-8	Cooked rice	1104	1106	7	0.01	3	0.00	6	0.02	8	0.02	8	0.02
3-Cyclohexene-1-methanol	1679-51-2	beer	1110	1106	2	0.02	0	/	0	/	0	/	0	/
2-Nonenal, (E)-	18,829–56-6	Cucumber、fresh	1157	1162	0	/	0	/	0	/	0	/	6	0.00
Pyrazine, 3,5-diethyl-2-methyl-	18,138–05-1	Paint	1159	1162	0	/	3	0.02	0	/	0	/	0	/
2,5-Dimethylbenzaldehyde	5779-94-2	Fishy、putrefaction	1226	/	7	/	0	/	2	/	5	/	8	/

#### 3.2.3 Comparative analysis of GC-O-QTOF and GC-IMS results

In the five scallop samples, 62 VOCs were identified by GC-IMS and 27 VOCs were identified by GC-O-QTOF, among which six compounds were co-detected, namely 2-nonanone, n-octanal, benzaldehyde, heptanal, hexanal and dimethyl sulfide. Hexanal had the highest content in five scallop samples and its threshold value was low, which significantly impacted the scallop's flavor and could be used as a key factor for evaluating the aroma of scallop. 56 compounds were detected by GC-IMS and not by GC-O-QTOF, presumably due to the low contribution of these compounds to scallop flavor. 21 compounds were detected only by GC-O-QTOF, and these 21 compounds may include some compounds that could not be characterized due to the incomplete GC-IMS database. As GC-IMS has higher sensitivity and faster detection speed, which makes it easier to detect more VOCs, the incompleteness of the database also makes GC-IMS incomplete in identifying compounds. In addition, GC-O-QTOF can identify more key aroma presenting compounds by aroma intensity, but the samples have to be pre-treated before they can be analyzed, which is not as easy and fast as GC-IMS. Therefore, the combination of GC-IMS and GC-O-QTOF can more comprehensively identify VOCs in scallops.

#### 3.2.4 Screening and analysis of key aroma compounds

To identify the key aroma compounds, we assessed the aroma intensities of 62 characteristic compounds using the time-intensity method (Table 2). Subsequently, 17 compounds with intensities ≥6 (GC-O ≥ 6) were identified and detailed in Table 1S. Among the 17 identified aroma compounds, some exhibited a fishy aroma, while others presented a pleasant, fruity freshness. 2-Acetyl-1-pyrroline in VFD and CAD had the largest aroma intensity value of 9, which could confer a certain rice aroma to VFD and CAD. Aroma compounds significantly influence the overall scent of scallops, with their impact gauged by the ROAV value. Generally, compounds with ROAV ≥1 were considered key contributors to scallop aroma. In this study, a total of nine aroma compounds with ROAV ≥1 were screened, as shown in Table 2S. 8, 5, 4, 5, and 5 key aroma compounds were screened in VFD, CAD, HAD, MWD, and ND, respectively. Dimethyl trisulfide was the most important contributor to VFD and HAD, with fishy and pungent odor. Dimethyl sulfide was the most important contributor to CAD, with a fishy odor, and Octanal was the most important contributor to MWD and ND, with a clear odor. It could be seen that VFD, CAD and HAD had a heavy fishy odor, while MWD and ND showed a certain fresh aroma.

**Fig. 4:**
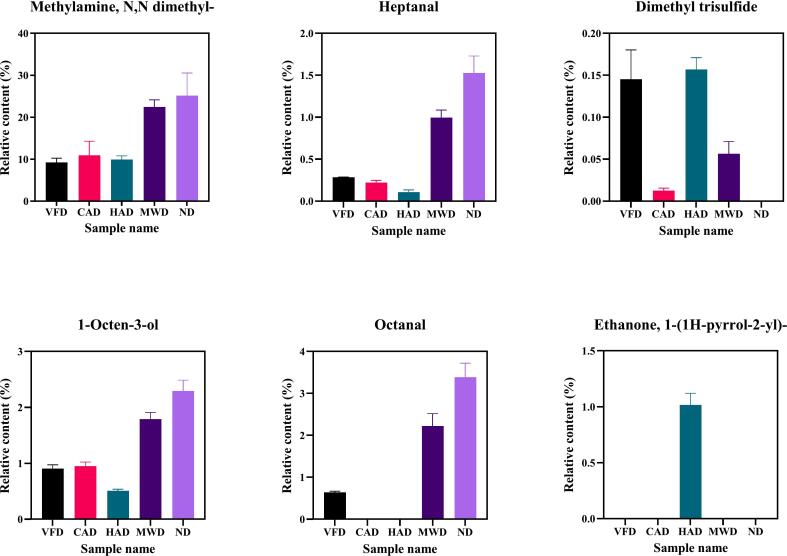
6 key aroma presenting compounds with GC-O ≥ 6 and ROAV ≥1.

In order to evaluate the key aroma presenting compounds more comprehensively, 6 differential compounds were further screened by the criteria of GC-O ≥ 6 and ROAV ≥1, as shown in Fig. 4. Methylamine, N, N dimethyl- (shredded squid), heptanal (shredded squid, fishy), 1-octen-3-ol (rust, paint), and octanal (fresh, fragrant) were significantly higher in ND than in the other four scallop species, suggesting that naturally dried scallops had a greater fishy odor. Dimethyl trisulfide was only unscented in ND and higher in VFD and HAD. The higher levels of Dimethyl trisulfide in hot air-dried scallops were attributed to the increased activity of sulfur-containing compound synthase during the heating process, resulting in the production of large amounts of sulfur-containing compounds. The low temperature of vacuum freeze-drying inhibited the loss of heat-sensitive sulfur-containing compounds and the degradation of macromolecular sulfur-containing compounds (Li et al., 2018). Octanal (Fresh, fragrant) was not sniffed in CAD and HAD, probably due to oxidative decomposition of octanal due to long drying time (Long et al., 2022). Ethanone, 1-(1H-pyrrol-2-yl)- was only smelled in HAD, giving the hot-air drying-treated scallops a plum aroma.

## 4 Conclusions

In summary, this study investigated the effects of five different drying methods, namely, natural drying (ND), hot air drying (HAD), vacuum freeze drying (VFD), microwave drying (MWD) and cold air drying (CAD), on the flavor of scallops. The characteristic flavor compounds in scallops were identified by HS-GC-IMS and GC-O-QTOF. 62 VOCs were clearly characterized by GC-IMS, among which the aldehydes had the highest content, which could reach 50 % - 70 % of the total content. The fingerprints of five types of scallops were constructed efficiently, and the differences of VOCs in the five types of scallops were distinguished visually. 27 characteristic aroma compounds were identified by GC-O-QTOF, and the highest content was trimethylamine. The intensity and odor of various aroma compounds were described by the time-intensity method, and 17 aroma compounds with GC-O ≥ 6 were screened, among which 2-Acetyl-1-pyrroline in VFD and CAD had the largest aroma intensity value of 9. The key 9 key aroma compounds were screened by ROAV ≥1. Finally, 6 differential compounds were further screened by combining the results of GC-O ≥ 6 and ROAV ≥1. This study provided a theoretical reference for the subsequent processing of scallops and the improvement of their flavor quality, and was of great significance for the identification of characteristic flavor compounds in scallops.

## CRediT authorship contribution statement

**Pengfei Jiang:** Writing – review & editing, Writing – original draft, Methodology, Formal analysis, Data curation, Conceptualization. **Xiaoqing Miao:** Writing – review & editing, Writing – original draft, Formal analysis, Data curation. **Jing Li:** Methodology, Investigation. **Hang Qi:** Visualization, Validation, Supervision, Software. **Shan Shang:** Visualization, Validation, Supervision, Software, Conceptualization. **Xiuping Dong:** Writing – review & editing, Validation, Resources, Funding acquisition, Conceptualization.

## Declaration of competing interest

The authors state that they have no known competing financial interests or interpersonal relationships that could have appeared to influence the work reported in this paper.

## Data Availability

No data was used for the research described in the article.

## Appendix A Supplementary data

Supplementary material Image 1
