# Supplementary material for: Volatile flavor characteristics of scallops (Chlamys farreri) with different drying methods were analyzed based on GC-IMS and GC-O-QTOF
Source: Food Chem X. 2024 Nov 1;24:101960. doi: 10.1016/j.fochx.2024.101960 (PMC11582753; doi:10.1016/j.fochx.2024.101960)
Supplement: Supplementary file 1 — Supplementary material [file mmc1.docx]

**Supporting Information**

compounds with intensity ≥ 6 (GC-O ≥ 6) in five scallops **(Table 1S)**

| No. | Compound | VFD | | CAD | | HAD | | MWD | | ND | |
| --- | --- | --- | --- | --- | --- | --- | --- | --- | --- | --- | --- |
|  |  | Strength | Relative content  (%) | Strength | Relative content  (%) | Strength | Relative content  (%) | Strength | Relative content  (%) | Strength | Relative content  (%) |
| 1 | Methylamine, N,N dimethyl- | 8 | 9.24 | 7 | 10.96 | 8 | 9.96 | 8 | 22.45 | 7 | 25.14 |
| 2 | Acetic acid | 4 | 0.87 | 5 | 2.19 | 6 | 2.62 | 1 | 0.53 | 4 | 1.03 |
| 3 | 2,3-Butanediol | 6 | 0.25 | 3 | 0.54 | 4 | 1.05 | 0 | / | 0 | / |
| 4 | Benzene, 1,3-dimethyl- | 0 | / | 0 | / | 0 | / | 6 | 0.52 | 5 | 0.28 |
| 5 | Heptanal | 5 | 0.28 | 7 | 0.22 | 5 | 0.11 | 7 | 0.99 | 6 | 1.53 |
| 6 | Pyrazine, 2,5-dimethyl- | 6 | 0.14 | 7 | 0.08 | 5 | 0.45 | 7 | 0.14 | 8 | 0.18 |
| 7 | 2-Acetyl-1-pyrroline | 9 | / | 9 | / | 8 | / | 5 | / | 6 | / |
| 8 | Dimethyl trisulfide | 7 | 0.15 | 2 | 0.01 | 5 | 0.16 | 3 | 0.06 | 0 | / |
| 9 | 1-Octen-3-ol | 8 | 0.9 | 5 | 0.95 | 3 | 0.51 | 7 | 1.79 | 5 | 2.3 |
| 10 | 5-Hepten-2-one, 6-methyl- | 8 | 0.17 | 7 | 0.04 | 6 | 0.03 | 5 | 0.03 | 4 | 0.03 |
| 11 | Octanal | 5 | 0.64 | 0 | / | 0 | / | 8 | 2.22 | 5 | 3.39 |
| 12 | Ethanone, 1-(1H-pyrrol-2-yl)- | 0 | / | 0 | / | 8 | 1.02 | 0 | / | 0 | / |
| 13 | Pyrazine, 2,6-diethyl- | 6 | 0.02 | 4 | 0.11 | 5 | 0.14 | 7 | 0.07 | 5 | 0.07 |
| 14 | Pyrazine, tetramethyl- | 4 | 0 | 2 | 0.1 | 8 | 0.1 | 6 | 0.08 | 6 | 0.02 |
| 15 | Ethanone, 1-(4,5-dihydro-2-thiazolyl)- | 7 | 0.01 | 6 | 0.02 | 3 | 0 | 8 | 0.02 | 8 | 0.02 |
| 16 | 2-Nonenal, (E)- | 0 | / | 0 | / | 0 | / | 0 | / | 6 | 0 |
| 17 | 2,5-Dimethylbenzaldehyde | 7 | / | 2 | / | 0 | / | 5 | / | 8 | / |

Note: Aroma intensity values refer to the sum of the total olfactory intensities.

ROAV of key flavor-presenting substances in five scallops **(Table 2S)**

| No. | Compound | Odour threshold  (μg/kg) | Flavor description | ROAV | | | | |
| --- | --- | --- | --- | --- | --- | --- | --- | --- |
|  |  |  |  | VFD | CAD | HAD | MWD | ND |
| 1 | Methylamine, N,N dimethyl- | 8 | Shredded squid | 23.10 | 30.14 | 23.66 | 89.8 | 65.99 |
| 2 | Dimethyl sulfide | 0.3 | Pale、Salty、Pyridine | 43.33 | 100.00 |  |  |  |
| 3 | Hexanal | 5 | Grass | 1.84 |  |  |  | 3.65 |
| 4 | Heptanal | 3 | Shredded Squid、Fishy | 1.87 | 1.61 | 0.70 | 10.56 | 10.71 |
| 5 | Dimethyl trisulfide | 0.03 | Fishy、pungent、air-dried | 100.00 | 7.33 | 100.00 | 64 |  |
| 6 | 1-Octen-3-ol | 1 | Rust、paint | 18.00 | 20.90 | 9.69 | 57.28 | 48.30 |
| 7 | Octanal | 0.7 | Fresh、fragrant | 18.29 |  |  | 100 | 100.00 |
| 8 | Ethanone, 1-(1H-pyrrol-2-yl)- | 3 | Snacks、plum、sweet |  |  | 6.46 |  |  |
| 9 | 2-Nonanone | 5 | Fragrant、neutral | 2.20 |  |  |  |  |
